# 

**Published:** 1921-09

**Authors:** 


					^O/V/AN
CONTENTS. Page
Remarks on Sinus Phlebitis and Thrombosis complicating Sup-
purative Middle Ear Disease.
By J. P. I. Hartv, B.A., M.B
B.Ch. (R.U.I.)- F.R.C.S.
73
Progress in the Treatment of Empyema.
By J. A. Nixon, C.M.G.
M.D. (Cantab.). F R.C.P.
82
^ Case of Multiple Serositis (Pick's Disease) of Unusual Dis-
tribution.
By H. J. Ork-Ewing, M.C., M.D., B.S. (Lond ), M.R.C.P.
go
reviews of Books
94
n8
Library of the Bristol Medico-Chirurgical Society   125
^bitu
ary:
Herbert Stanley Ballance
127
^-ocal Medical Notes
Editorial Notes
128

				

